# Adolescent HIV disclosure in Zambia: barriers, facilitators and outcomes

**DOI:** 10.7448/IAS.17.1.18866

**Published:** 2014-03-10

**Authors:** Gitau Mburu, Ian Hodgson, Sam Kalibala, Choolwe Haamujompa, Fabian Cataldo, Elizabeth D Lowenthal, David Ross

**Affiliations:** 1 International HIV/AIDS Alliance, Hove, UK; 2 Division of Health Research, Lancaster University, UK; 3 Center for Global Health, Trinity College Dublin, Ireland, UK; 4 Population Council, Washington, DC, USA; 5 Alliance Zambia, Lusaka, Zambia; 6 Research Department, Dignitas International, Zomba, Malawi; 7 Departments of Pediatrics and Epidemiology, Perelman School of Medicine, University of Pennsylvania, PA, USA; 8 Children's Hospital of Philadelphia, PA, USA; 9 Department of Infectious Disease Epidemiology, London School of Hygiene and Tropical Medicine, London, UK

**Keywords:** adolescents, sexuality, HIV, disclosure, Zambia, Africa

## Abstract

**Introduction:**

As adolescents living with HIV gain autonomy over their self-care and begin to engage in sexual relationships, their experiences of being informed about their HIV status and of telling others about their HIV status may affect their ability to cope with having the disease.

**Methods:**

In 2010, we conducted a qualitative study among adolescents aged 10–19 living with HIV in Zambia, and with their parents and health care providers. Through interviews and focus group discussions, we explored the disclosure of HIV status to adolescents living with HIV; adolescents’ disclosure of their status to others; and the impact of both forms of disclosure on adolescents.

**Results:**

Our study identified three main barriers to disclosure of HIV status: local norms that deter parents from communicating with their children about sexuality; fear of HIV stigma; and an underlying presumption that adolescents would not understand the consequences of a HIV diagnosis on their lives and relationships. With regard to adolescents’ disclosure of their HIV status to their sexual partners, our study identified fear of rejection as a common barrier. In rare cases, open family conversations about HIV helped adolescents come to terms with a HIV diagnosis. Findings indicated that disclosure had various outcomes at the individual and interpersonal levels. At the individual level, some adolescents described being anxious, depressed and blaming themselves after being told they had HIV. At the interpersonal level, disclosure created opportunities for adolescents to access adherence support and other forms of psychosocial support from family members and peers. At the same time, it occasionally strained adolescents’ sexual relationships, although it did not always lead to rejection.

**Conclusions:**

There is a need for public health interventions that guide adolescents living with HIV, their parents and families through the disclosure process. Such interventions should help parents to assess and understand the evolving cognitive capacity and maturity of their adolescents in order to determine the appropriate time to inform them of their HIV-positive status. Such interventions should also mitigate the risk of HIV stigma, as well as local norms that may prevent discussions of sexuality within families. Adolescents who have been informed of their HIV status should be provided with on-going support to prevent disclosure from negatively affecting their psychological and sexual wellbeing. Further research is needed to explore the potential role of trusted family members in contributing to the disclosure process.

## Introduction

An estimated 2 million adolescents (aged 10–19 years) are living with HIV worldwide, most of whom are unaware of their HIV status [1]. Many of these adolescents are also engaging in romantic and sexual relationships [2, 3]. In this context, the importance and potential effects of disclosure of an adolescent's HIV-positive status to him/her, and of disclosure by the adolescent to others, have drawn the interest of researchers. Recent studies have sought to identify patterns, models, barriers and consequences of disclosure among adolescents living with HIV [4, 5].

The literature on HIV disclosure describes four distinct scenarios relating to adolescents: one is when parents and guardians disclose their own HIV-positive status to their children [6]; the second is when parents, guardians or other caregivers, such as health care providers, inform adolescents living with HIV that they (adolescents), are HIV-positive [4]; the third is when adolescents living with HIV disclose their status to a third party [7, 8]; and the fourth is when adolescents’ HIV-positive status is disclosed to a third party by their parents, guardians or health care providers [7].

Each of these forms of disclosure, when planned and timed well, can be beneficial. For instance, parents who disclose their own HIV-positive status to their children may experience better relationships with them [6]. In addition, adolescents who have been told about their own HIV-positive status may be in a better position to access antiretroviral therapy (ART) from health facilities and psychosocial support from peer support groups [9]. At the same time, explaining to adolescents that they have HIV fulfils their right to know about their own health [10, 11], and it may also improve their adherence to ART [12], retention in HIV care [13] and subsequent survival [14]. Additionally, it may boost their self-esteem [15], help them cope more effectively with stigma [9] and encourage them to adopt sexual behaviours that could protect their health and the health of sexual partners [16, 17]. As for adolescents determining who else knows about their HIV-positive status, the experience of choosing who should know, and choosing when and how to disclose to them, may help adolescents feel more empowered and autonomous [18].

However, disclosure of adolescents’ HIV-positive status is a delicate undertaking that may have negative consequences. Studies that examine HIV disclosure among young people suggest that disclosure ought to be a *process* rather than a single event [19, 20]. Current global guidelines [21] from the World Health Organization (WHO) recommend disclosure to young people once they are of school age. The guidelines recommend that younger children be informed of their own and their parents’ or caregivers’ HIV-positive status incrementally in accordance with their growing cognitive skills and emotional maturity. In practice however, disclosure to adolescents living with HIV in sub-Saharan Africa varies widely and is often inconsistent with these recommendations.

For instance, parents living with HIV are frequently reluctant to disclose their own sero-status to adolescents, and they often delay informing adolescents of the adolescents’ HIV infection [22, 23]. In a recent study in the Democratic Republic of Congo, one-third of caregivers of HIV-positive children aged 5–17 saw no benefit in informing them that they were infected with HIV [24]. Although some parents may plan to disclose this information at some point in the future [24], in practice, informing HIV-positive adolescents about their HIV status is frequently unplanned [25], and often tends to be a single event rather than an open and on-going discussion [26]. In other cases, adolescents’ HIV status is disclosed to third parties without the adolescents’ consent, involvement or awareness, leading to negative reactions from adolescents [7, 18]. These patterns of disclosure can have a detrimental impact on how adolescents cope with the knowledge that they are living with HIV.

Understanding the process of disclosure and its consequences for adolescents in different contexts is critical for determining whether, how and when disclosure should take place, as well as for identifying what types of support adolescents and parents need in order for disclosure to be beneficial. In this paper, we document the experiences of adolescents living with HIV with regard to disclosure, specifically addressing the second and third scenarios identified earlier: adolescents who were previously unaware of their HIV-positive status being told about it by their parents, and adolescents who know about their HIV-positive status telling others about it. In particular, we explore the following three questions: first, what barriers to disclosure were encountered; second, once adolescents were aware of their HIV-positive status, what factors influenced their decision to disclose it to others; and third, what impact did disclosure have on the adolescents?

## Methods

### Study setting

Adolescents aged 10–19 make up almost one-fourth of Zambia's population of 13 million [27]. According to the most recent demographic and health survey estimates, HIV prevalence in Zambia is 14.3% among the general population and 4.7% among those aged 15–19 [28]. At the end of 2009, UNICEF estimated that 80,000 adolescents aged 10–19 were living with HIV in Zambia [29]. Modelling data suggest that the absolute number of HIV-positive adolescents in southern Africa maybe on the rise due to improved adolescent access to ART and subsequent better survival [30].

In this paper, we report previously unpublished findings from a study documenting the experiences of Zambian adolescents living with HIV conducted at one rural site and two urban sites across Zambia: Kalomo (a rural site in the Southern Province), Kitwe (an urban site in the Copperbelt Province) and Lusaka (the national capital). HIV prevalence varies widely among young people aged 15–24 in these locations: it is 3.3% in the Southern Province, 4.8% in the Copperbelt Province and 6.6% in Lusaka [28]. We recently reported that the health systems in the three study areas were facing challenges in meeting the sexual and reproductive health needs of adolescents living with HIV [25] at a time when adolescents’ sexual autonomy and expressiveness were increasing [18].

The above study sites were selected to represent the experiences of adolescents living with HIV in diverse settings, as social context greatly determines the experiences of this population [31, 32]. At all three sites, adolescents living with HIV were accessing ART and other clinical services at government facilities. Typical services provided to adolescents included clinical consultations, HIV testing and counselling, prescription of ART, and screening and treatment for opportunistic infections. Adolescents in our study were also accessing non-clinical services provided by community-based organizations and youth centers run by non-governmental organizations (NGOs). These services included nutrition support, youth-led peer support, adherence and psychosocial counselling, home visits and other community outreach interventions.

### Study participants and recruitment

All study participants were recruited through government ART clinics or within neighbouring community and youth centers run by NGOs. Researchers visited these clinics and centres and provided an overview of the study to the facility staff. Subsequently, adolescents were invited to participate in the study, together with their parents and some of their health care providers. The study enrolled three groups of participants: adolescents aged 10–19 living with HIV, regardless of mode of HIV acquisition; their parents and guardians; and their health care providers. A greater number of adolescents were enrolled compared to other study participants because the primary purpose of the study was to explore adolescents’ own perceptions of their experiences; data from the adult groups were used for triangulation purposes. All adolescents in the study were aware of their HIV status and were already accessing services.

### Data collection

Qualitative data were collected through interviews and focus group discussions. Combining both methodologies provided an opportunity for complementary information to be obtained, considering that the interactive nature of focus groups can provide detailed information based on the diversity of participants’ experiences. Interviews and focus group discussions were conducted by a team of researchers (two males and one female) experienced in qualitative data collection. These researchers received additional training on adolescent-specific interviewing techniques and on ethical issues. Interviews and focus group discussions were held at HIV clinics or community centers, and in the case of adolescents, they were held in the presence of parents or guardians. Participants, especially adolescents, were asked to choose a place that offered them convenience and privacy. As adolescent participants were enrolled in the study, they were assigned either to focus group discussions or interviews on an alternating basis to ensure adequate participation in both methodologies. All data collection took place between April and December 2010.

#### Interview methodology

Researchers used semi-structured questionnaires to separately interview adolescents and health care providers. Interviews with adolescents focused on their sexual and reproductive needs, and on their experiences of disclosure. Interviews with health care providers explored how the health services were meeting those needs and how adolescent disclosure could be supported and improved. Slightly different versions of the questionnaire were tailored to younger adolescents (10–14 years), older adolescents (15–19 years) and health care providers. Interviews were conducted in English or in a local language (Bemba, Lozi or Nyanja). All interviews lasted 30–40 minutes and were audio-recorded.

#### Focus group discussion methodology

Eight focus group discussions were held with a total of 53 adolescents, two with 21 parents of mixed gender (one urban group and one rural group), and three with 24 health care providers of mixed gender (two urban groups and one rural group). The adolescent groups included two with girls aged 10–14, two with girls aged 15–19, two with both boys and girls aged 10–14 and two with both boys and girls aged 15–19. The focus group discussions used open-ended questions and follow-up probes to explore how health services were meeting the needs of adolescents living with HIV; adolescents’ adherence to ART; and the social contexts of adolescents and their experiences of disclosure. Participants’ preferences determined whether the focus group discussions took place in English or a local language. All discussions lasted 50–60 minutes and were audio-recorded, with the researchers taking additional notes.

### Data analysis

Interview and focus group data were transcribed and translated into English, then were encoded in QSR International's NVivo 7 [33]. Separate thematic content analyses were performed on the two bodies of data consistent with common practice [34]. Codes were organized into basic themes and subsequently re-organized hierarchically in an iterative process guided by the study objectives. Two authors (GM and IH) independently analyzed the data, checked for variations in interpretation and reconciled such divergences.

### Ethical considerations and approval

The study was conducted in accordance with provisions of the Declaration of Helsinki [35], and privacy and confidentiality were safeguarded consistent with guidelines for research involving young people [36]. Comprehensive information was provided to adolescents, their parents or guardians and health care providers in their own languages. Researchers also confirmed in advance with parents and health care providers that all adolescents approached about the study already knew their HIV status [11]. Parents and guardians signed consent forms for orally assenting adolescents aged 10–18, while adolescents aged 18–19 signed their own consent forms in the presence of their parents. (The legal age of adulthood in Zambia is 18). Parents and health care providers signed their own consent forms. The study received approval from the Biomedical Research Ethics Committee of the University of Zambia.

### Data presented in this paper

In this paper, we present previously unpublished data related to disclosure, focusing on barriers and outcomes of disclosure. We identify by subheadings the most prominent themes to emerge from our analyses of the data, and provide a narrative account using illustrative quotes.

## Results

### Characteristics of study participants

A total of 58 adolescents living with HIV participated in the interviews, while 53 adolescents living with HIV participated in the focus group discussions. All adolescents were aware of their HIV status, and they were accessing clinical and non-clinical services. Six older adolescents in the study were married and had children of their own. Health care providers, who were drawn from both governmental HIV clinics and non-governmental and community-based organizations, included medical doctors, nurses, counsellors and administrative staff. Health care providers participated in interviews (*n=*14) and focus group discussions (three sessions; *n=*24). Parents participated in only focus group discussions (two sessions; *n=*21). More details are presented in Table 1.

**Table 1:** Characteristics of study participants

	Interviews	
	
	*Adolescents*	*Health care providers*
Age range	10–19	32–47
Mean age	16.8	36
Rural	12 (21%)	5
Urban	46 (79%)	9
Male	29	5
Female	29	9
In sexual relationship	29 (50%)	–
Planning to have children	28 (48%)	–
Total	58	14
	**Focus group discussions**	
*Adolescents living with HIV*		
	Female sessions	4 sessions; *n=*18
	Mixed sessions	4 sessions; *n=*35 (female=48.6% in mixed sessions)
	Urban	22 (42%)
	Rural	31 (58%)
	Total	8 sessions; *n=*53
*Health care providers*		
	Urban	2 sessions; *n=*16
	Rural	1 session; *n=*8
	Total	3 sessions; *n=*24
*Parents and guardians*		
	Urban	1 session; *n=*8
	Rural	1 session; *n=*13
	Total	2 sessions; *n=*21

Qualitative data analysis identified a range of interrelated factors associated with the experiences of adolescents living with HIV with regard to disclosure of their HIV status to them, adolescents’ disclosure to others and the outcomes of disclosure on adolescents. These factors and their implications for HIV programming and research are described in Table 2. Across all of the data, including data from same-sex focus group discussions, there were no apparent thematic differences in the disclosure experiences of male and female adolescents. However, older adolescents generally provided more detailed accounts of their disclosure experiences. Adolescents seemed to be more forthcoming among peers in the focus group discussions than they were in individual interviews.

**Table 2:** Emerging themes and implications for programming and future research

Issue	Coding concepts	Broad emergent themes	Implications for programming and research
Informing adolescents of their HIV-positive status	Adolescents’ understanding of HIV	Adolescent evolving capacity	Parents may need training on how to recognize adolescents’ evolving capacity and level of understanding
	Parental willingness or reluctance to disclose	Stigma	Interventions needed to support families in having open communication about sexuality
	Social/cultural environment: stigma, cultural norms, taboos, traditions	Social norms	Future studies should explore the role of other trusted adults, apart from parents, in the disclosure process
Adolescent disclosure to others	Adolescents’ control over disclosure	Adolescent self-determination	Interventions to support disclosure should also address adolescents’ social environments including HIV stigma
	Social environment: stigma, fear of rejection by sexual partners	Stigma
	Interpersonal relationships: family and peers	Connectedness to family and peers	
Impact of disclosure	Adolescent reactions to disclosure: anxiety, depression and self-blame	Mental wellbeing	Adolescents who have been informed of their HIV status require on-going support to overcome negative outcomes of disclosure
	Reaction of sexual partners of adolescents: acceptance or rejection	Sexual wellbeing	
	Outcomes at the family and peer level	Treatment adherence and psychosocial support	

### Informing adolescents about their HIV-positive status

A strong theme that emerged from focus group discussions with parents and health care providers was the difficulty of determining when adolescents should be told about their HIV-positive status. Parents repeatedly spoke of planning to disclose to adolescents in the future, but many parents did not state how soon or under what circumstance they planned to disclose:

At what age should we start talking about these things? Yes, I feel that we should talk to them, but when should we start? (Focus group discussion, parents, Lusaka)

Many health care providers also thought that adolescents would be too young to understand what it means to have HIV:

Another challenge is you don't know at what age children will understand [HIV] because some mature quickly and others don't. So you could be talking about HIV and they don't know what it is and … don't understand what is affecting them. (Interview, NGO-based counsellor, Lusaka)

The younger ones depend on their parents, but when they get to [age] 10, they need to start understanding what's happening to them. But maybe they do not [understand]. That is a challenge. (Interview, hospital-based counsellor, Lusaka)

Comments from adolescents suggested that parents were reluctant to enter into disclosure conversations and that some disclosure experiences may have resulted from the adolescents’ inquisitiveness:

I used to come to the hospital to [collect] medicine without knowing the reason why, until I finally confronted my father and he told me the truth … My mother did not want to tell me. Every time I asked her what was wrong with me, she would not tell me. (Interview, 14-year-old female, Lusaka)

This reluctance among parents appeared to be related to HIV stigma, which was not uncommon in the study setting. Among parents who were themselves living with HIV, stigma imposed a double burden: parents who had not come to terms with their own HIV status were likely to find it even more difficult to disclose to their children about the children's HIV infection. In the words of a nurse interviewed in Lusaka, “A parent needs to accept their own status before they can start talking about [their child's HIV] status.”

Local norms relating to sexuality also appeared to discourage parents from telling their adolescent children that they had HIV. According to one parent participating in a focus group discussion in Lusaka, “Sexuality cannot be discussed openly between parents and children.” Another parent in the same discussion emphasized, “As a father, you can't talk to your daughter about sex and so on. If a father talks to a child about such things, then he has no respect.” The centrality of cultural norms in determining the content of conversations between parents and their children was also illustrated by health care providers:

Well, it's against our tradition: it is not allowed for parents to talk their children about matters of sexuality. (Focus group discussion, nurse, Lusaka)

Even in the community, it's a taboo. You cannot just discuss such issues. (Focus group discussion, clinic administrative staff, Lusaka)

### Adolescents’ disclosure to others

Adolescents living with HIV expressed a desire to control whether and to whom to disclose their HIV status:

Disclosure is something you choose to do. It's your choice whether to tell people about it. You could decide that you are the only person who will know about it. It's a choice that people [should] make. (Interview, 18-year-old male, Lusaka)

However, adolescents did not always have a choice regarding who knew their status. Household members were almost always aware of adolescents’ HIV status, sometimes as a necessity:

How could I have hidden that from my sister when she is the one who even took me to the family unit at the HIV clinic, after I was diagnosed HIV-positive? (Interview, 18-year-old male, Lusaka)

Another issue that adolescents discussed in relation to disclosure was stigma. Asked if they had told friends at school about having HIV, adolescents indicated that disclosure at school was rare. Some described their efforts to maintain secrecy:

There is no one who knows [my status]. When they find me at the clinic, I just tell them I have escorted somebody else. (Interview, 15-year-old female, Kitwe)

My biggest challenge has been at school, because usually a lot of people make fun of me. They come to me and say: “You, we know you are sick with something.” (Interview, 16-year-old male, Lusaka)

An additional barrier relating to adolescent disclosure was fear of abandonment by romantic or sexual partners. Adolescent girls interviewed who had not disclosed their HIV status to their boyfriends cited the fear that disclosure could end their relationships:

I have a boyfriend at the moment but it is not a sexual relationship. He does not know about my status. I am scared of telling him because he might leave me. (Interview, 19-year-old female, Lusaka)

Although in our sample the number of adolescents who had not yet disclosed their sero-status specifically to sexual partners was low (*n=*2 out of 27 adolescents in sexual relationships), health care providers noted that such a situation presents a difficult dilemma:

This is a huge challenge for the adolescents because about half of the girls and boys who come here are in relationships with [partners] who are HIV-negative, and they don't want to disclose their status for fear of being dumped. (Focus group discussion, hospital-based nurse, Lusaka)

While these barriers existed, the connectedness that adolescents felt with their friends at community centres influenced the ways in which disclosure took place, showing that interpersonal relations play an important role in self-disclosure. Despite the significant amount of time that adolescents spent at school, there was limited disclosure to peers in school settings:

When I found out that I was sick, my relationships with my friends did not change because most of my friends whom I play with do not know about my [HIV] status. I have not told them. Only my friends at the [community] centre know. (Interview, 16-year-old female, Lusaka)

My friends at school do not know that I am HIV-positive, but everyone at home knows. (Interview, 15-year-old female, Lusaka)

### Impact of disclosure

Three themes emerged from our data relating to the impact of disclosure on adolescents’ emotional wellbeing, sexual relationships and adherence. Adolescents reacted to disclosure with varying degrees of anxiety, depression and withdrawal:

When I first found out that I was HIV-positive, I was very depressed. I wanted it to be a family secret and only disclose it when I felt that I was ready, and only to people I felt I could trust. (Focus group discussion, 15–19-year-old females, Lusaka)

Counselling was difficult for me because it was very hard for me to accept my status. I started taking my [antiretroviral medication] last week and I am still finding it difficult, especially swallowing the pills. I actually throw up. (Focus group discussion, 15–19-year-old females, Lusaka)

There were instances in which adolescents initially internalised blame, but thereafter adjusted positively:

When I knew, I was like, “Why me?,” and … blaming myself, almost extending it to God. But now I am okay. I'm like: “It's nature.” It's not my decision to be in such a state, and so if I take my drugs no one will notice. I would be a normal child just like any other. So I am like, “It's just part of life.” (Interview, 15-year-old male, Lusaka)

It appeared that relatives had the potential to contribute to the disclosure process in ways that helped adolescents cope with learning about their HIV status. In one case, an adolescent reported being asked by his uncle on multiple occasions to consider what it would be like to receive a diagnosis of HIV. The uncle's approach appears to be consistent with the concept of making disclosure a process rather than a single, discrete event:

I wasn't very shocked; I took it like a normal person because we used to talk about it with my uncle. I used to hang out with my late uncle and he used to ask me how I would take it if I learnt that I was HIV-positive. At first I was like: “I would not take it,” but he would say that [he] will also go and get tested. So eventually I stopped getting scared. (Interview, 18-year-old male, Lusaka)

In addition to affecting their emotional wellbeing, adolescents’ disclosure experiences were associated with varying consequences for romantic relationships. Although many study participants anticipated and feared being rejected, the study found little empirical evidence that this occurred. In a few cases where the loss of relationships was reported after disclosure, this was often not a direct consequence of disclosure itself:

I only disclosed my status to one of my ex-boyfriends. He understood me because we never had sex and we went out for a long time. Eventually he started asking me for sex and we broke up. (Interview, 19-year-old female, Lusaka)

As noted in the above quotation, romantic partners often responded well to disclosure because “It is possible to have a normal relationship even though you are positive,” as a 19-year-old female interview participant stated.

Another outcome of disclosure was antiretroviral adherence support, especially from family members. Many adolescents discussed how vital the adherence support from family members was to them in their efforts to take medications as required:

On my own, I don't think I would manage [to adhere]; I need a lot of help from loved ones who know my [HIV] status. (Interview, 17-year-old male, Kalomo)

I do not find taking medicine to be a difficult task any more. I easily follow my treatment regime. My auntie always reminds me to take my medicine. (Interview, 15-year-old female, Lusaka)

Much less frequently, support from friends was also described:

Yeah, some of my close friends know that I am sick. They encourage me to be taking the medicine so that I can be okay and live longer. (Interview, 18-year-old female, Kitwe)

Taken together, these quotes suggest that adolescents’ self-disclosure to others may create a social space for much-needed psychosocial and adherence support.

## Discussion

Although there is no consensus on how or when children should be told that they are living with HIV [20], recent studies suggest that disclosure should take place by the time they reach adolescence [11, 37, 38] and before they become sexually active [4]. This is consistent with the recommended approach of providing young people with information about their HIV status in accordance with their developmental capacity, leading to full disclosure once they have sufficient cognitive and emotional capacity to understand the meaning of an HIV diagnosis [21, 39, 40]. Our study findings are consistent with other studies suggesting that disclosure can open up avenues for adherence and psychosocial support [9, 12, 41]. Similar to other studies, it also documents barriers to telling adolescents about their HIV-positive status, such as believing the child is not ready or is too young to comprehend the meaning of an HIV diagnosis [4, 11, 23, 42], as well as fear of stigma and discrimination [5, 41].

Our study adds to this literature by showing that the link between HIV and sexuality presents a barrier to disclosure in Zambia, where local norms make it difficult for parents to discuss sexuality with their children. According to health care providers in our study, parents’ reluctance to inform adolescents that they (the adolescents) were infected with HIV was related to the parents’ perception that disclosure of HIV status was inextricably linked to discussions of sexuality, and therefore could not be done without violating cultural conventions.

Other studies have shown that in sub-Saharan Africa, the boundaries of parent-to-adolescent communication about sexuality are often delineated by societal norms and taboos [43]. In a recent systematic review of factors that shape young people's sexual behaviour, Marston and King show that social expectations determine communication about sex more generally, regardless of whether or not HIV is a consideration [44]. When we consider this finding in relation to existing recommendations on HIV disclosure to adolescents [21], it raises important concerns about the extent to which these recommendations can be implemented, given the link between HIV and sexuality. The challenge is further compounded by poor parental knowledge of sexual and reproductive health in our study setting and elsewhere in sub-Saharan Africa – a factor shown to hinder parent–adolescent communication [18, 43, 45]. Other studies have shown that poor parent–adolescent communication affects disclosure of parents’ and adolescents’ HIV status to adolescents [24, 46, 47]. These findings indicate a need to improve the sexual communication skills of parents in order to facilitate disclosure. Open communication in a family, whether about HIV or other issues, could improve disclosure experience and emotional outcomes for adolescents [46].

Part of the difficulty parents face in telling their adolescents that the adolescents have HIV is related, we propose, to parents’ discomfort around the issue of their own HIV status. Although our study did not specifically seek to enrol parents who were living with HIV, our findings suggest that HIV-positive parents who had not come to terms with their own status tended to find it more difficult to inform an adolescent son or daughter that he or she also had HIV. Other studies have shown that parents who are more open about having HIV themselves are more likely to tell their adolescents about the adolescents’ own HIV status [40, 42, 48]. This finding supports the conclusion from a recent systematic review exploring disclosure of children's HIV status to children themselves [39]. High levels of internalised HIV stigma and shame on the part of the parents living with HIV can interfere with adolescent disclosure [49]. These observations suggest a need for HIV programs to support HIV-positive parents to come to terms with their own diagnosis through HIV counselling and education, both for their own benefit and so that they can confidently discuss HIV-related information with their adolescent children [24, 50, 51].

Our findings also suggest that complete reliance on parents and health care providers as the primary sources of disclosure to adolescents, as suggested by caregivers in South Africa and India [4, 52], may not be ideal. In our study, there was an instance in which an uncle played a role in preparing an adolescent for disclosure. This suggests that open and caring adults, whom adolescents trust, could assist with disclosure and could be a source of psychosocial support following disclosure. It also suggests that adolescents’ reactions to HIV disclosure might be influenced by open conversations around HIV with relatives or other adults as part of a disclosure *process*. However, before conclusions can be drawn about the supportive role that relatives might play, further studies are needed to explore how family-centred approaches may facilitate parent-to-child disclosure, bearing in mind that health care providers may also have contributions to make to this process [53].

Another dimension of our findings relates to the issue of adolescents disclosing their HIV-positive status to others. Research from a range of different settings suggests that HIV-positive adolescents are often reluctant to disclose their HIV status to their romantic and sexual partners, mainly for fear of rejection and stigmatization [5, 7, 54, 55]. Because our study was conducted among adolescents who were HIV-positive and knew their status, the reluctance of a few of them to disclose their status to their sexual partners raises concerns about onward sexual transmission of HIV. Recent studies examining disclosure among HIV-positive youth support the assertion that non-disclosure among adolescents could undermine HIV prevention [2, 8]. This is notable given that disclosure to sexual partners is a particularly challenging form of disclosure [56], and as a result, adolescents are often hesitant to disclose to casual sexual partners [16]. These findings suggest that adolescents may need support in understanding benefits of disclosure, and in making decisions relating to if, when, and how to disclose, and to whom, including in relation to their sexual partners [8]. Such interventions should respond to adolescents’ evolving capacity and sexual autonomy [18, 19], and to the need to promote the health of adolescents living with HIV and their sexual partners [2].

Finally, our study illustrates that disclosure outcomes vary among adolescents in relation to their social context, ranging from short-term anxiety and depression, as well as impact on sexual relationships and on avenues for adherence and other psychosocial support. Our study findings are particularly important in this regard, given that few studies document the impact of disclosure on children [39]. The short-lived emotional impact described by adolescents in our study is consistent with findings in western contexts showing that disclosure does not seem to have major long-term negative psychological outcomes [56, 57], but may cause short-term anxiety [46, 58]. These events are relevant given that fear of negative psychological consequences may be a key reason for parents' own failure to tell young people about their (young people's) HIV-positive status [23, 59–61]. These findings also suggest a need for interventions that could enable parents and adolescents to recognize and respond to these negative psychological consequences. For instance, improving parental and health care provider communication with young people before, during and after disclosure, as suggested by Vaz et al. [26], could help adolescents adjust to the news that they have HIV. The availability of ART in Zambia and other settings could also enable positive messaging regarding the meaning of an HIV diagnosis and the health prognosis for adolescents, which could further ameliorate potential emotional difficulties following disclosure. Evidence suggests that disclosure to adolescents is more likely if they are on ART [48].

### Strengths and limitations

This paper provides evidence that HIV disclosure to HIV-positive adolescents in Zambia is delayed by fear of HIV-related stigma, a belief among parents and health care providers that adolescents may not fully comprehend the meaning of an HIV diagnosis, and cultural norms that prohibit discussions on sexuality between parents and their children. On their part, adolescents in our study were hesitant to disclose their HIV status to romantic and sexual partners for fear of being rejected, which raises concerns in relation to HIV prevention. While our findings may not be generalizable to all settings, they can inform adolescent programming.

In particular, our study identifies potential opportunities to improve the process of disclosure, as illustrated in Table 2. Practical interventions to build communication between parents and their adolescent children about sexuality could make it easier for parents to inform adolescents of their HIV-positive status. HIV programs could incorporate community-based interventions that challenge or transform social–cultural values related to how families discuss sexuality. This is consistent with an approach that considers the wider social environment of adolescents and acknowledges that the experience of adolescents are influenced by factors located at the individual, family, peer, community and structural levels in determining relevant interventions [31].

Our findings also suggest that efforts to reduce HIV stigma in the community could have a beneficial effect on the process of informing adolescents that they are HIV-positive, as suggested by others [41]. We further speculate that reduced levels of stigma could encourage adolescents to disclose to their sexual partners, since the main reason why adolescents in our study did not disclose their status to their romantic partners was fear of rejection on the basis of their HIV status.

While the World Health Organization recommends an incremental approach to informing children of their own and their parents’ or caregivers’ HIV status in accordance with the children's growing cognitive skills and emotional maturity [21], our study suggests a need to train parents to better understand the evolving cognitive capacity of young people to understand the meaning of a positive HIV diagnosis and to better recognize when adolescents are ready to be told about their status. Our study also identified an important area for further research, related to the potential role of trusted family members in supporting disclosure. More broadly, as recommended by others [52, 59, 61], local, context-specific and culturally sensitive disclosure guidelines, models and tools are urgently needed.

While our study did not include adolescents who had not been informed of their HIV-positive status, and therefore did not estimate the prevalence of disclosure, our findings suggest that for those adolescents who have been informed of their HIV status, providing them with on-going psychosocial support and counselling in relation to sexual health is required to mitigate the negative consequences of disclosure on their psychological and sexual wellbeing. While it is possible that some of the negative psychological outcomes of disclosure may improve over time, we did not have adequate data to support that conclusion because our cross-sectional study only provides a snapshot of adolescents’ perceptions at one point in time. Since disclosure is evolving process [20], a longitudinal study design would be more suited to detect changes in how adolescents perceive and experience disclosure over time. Although our study shows that disclosure opened up avenues for adolescents to receive adherence support from family and peers, the study was not designed to quantitatively measure the impact of disclosure on adherence. Nonetheless, positive impact of disclosure on adherence has been reported in other studies [12, 62].
